# Roles of Ependymal Cells in the Physiology and Pathology of the Central Nervous System

**DOI:** 10.14336/AD.2022.0826-1

**Published:** 2023-04-01

**Authors:** Shiyu Deng, Lin Gan, Chang Liu, Tongtong Xu, Shiyi Zhou, Yiyan Guo, Zhijun Zhang, Guo-Yuan Yang, Hengli Tian, Yaohui Tang

**Affiliations:** Shanghai Jiao Tong University Affiliated Sixth People’s Hospital, School of Biomedical Engineering, Shanghai Jiao Tong University, Shanghai 200030, China.

**Keywords:** Ependymal cells, central nervous system, CNS development, CNS disease

## Abstract

Ependymal cells are indispensable components of the central nervous system (CNS). They originate from neuroepithelial cells of the neural plate and show heterogeneity, with at least three types that are localized in different locations of the CNS. As glial cells in the CNS, accumulating evidence demonstrates that ependymal cells play key roles in mammalian CNS development and normal physiological processes by controlling the production and flow of cerebrospinal fluid (CSF), brain metabolism, and waste clearance. Ependymal cells have been attached to great importance by neuroscientists because of their potential to participate in CNS disease progression. Recent studies have demonstrated that ependymal cells participate in the development and progression of various neurological diseases, such as spinal cord injury and hydrocephalus, raising the possibility that they may serve as a potential therapeutic target for the disease. This review focuses on the function of ependymal cells in the developmental CNS as well as in the CNS after injury and discusses the underlying mechanisms of controlling the functions of ependymal cells.

## 1. Introduction

Ependymal cells are neuroepithelial multiciliated cells lining the spinal cord and cerebral ventricles [1], and are derived from radial glial cells in the embryo between embryonic Day 14 (E14) and E16 [2]. They are born first as nonmotile monopiled epithelial cells and then mature as motile multiciliated cells during the first two postnatal weeks. Immature ependymal cells protrude short and randomly oriented cilia into the brain ventricles. During maturation, the cilia length increases, starts beating, and produces a fluid flow that alters the basal bodies in the same direction. A recent study showed that mature ependymal cells can be detected at postnatal day (P0) as mature state-related genes (including Lrcc1, Meig1, Foxj1, etc.) are already highly expressed [3]. However, the mechanisms that control ependymal cell differentiation and maturation have not been fully revealed. It is known that some transcription factors are necessary for regulating the development of ependymal cells. Retinal ganglion cells can express transcription factors GemC1 and Mcidas, and both participate in ependymal cell maturation by activating their downstream transcription factors such as Foxj1 and Myb [4]. Foxj1 is involved in regulating cilia formation and epithelial morphology during ependymal cell maturation. Without Foxj1, ependymal cells fail to differentiate from radial glial cells [5]. Lavado et al. reported that the homeobox gene Six3 is expressed in ependymal cells during lateral ventricle formation, and deletion of Six3 resulted in the failure of ependymal cell maturation, lateral wall deficits, and hydrocephaly. Similarly, Myb is essential for modulating the differentiation of ependymal cells and cilia formation [6]. Recent studies have found that the transcription factor nuclear factor IX (NFIX) regulates ependymal cell maturation by controlling Foxj1. NFIX deficiency in mice results in aberrant ependymal cell morphology as well as aberrant thickening and loss of the ependymal cell layer within the postnatal brain [7].

Some investigators have suggested that multiciliated ependymal cells lack proliferation in the normal adult mouse brain [2]. However, in some cases, they still possess the ability to proliferate or regenerate, and limited repair capacity was detected under certain pathological conditions. In the aging brain, the ependymal repair is at a moderate level, and ependymal cells become elongated, extend long, and from tangled cilia [8]. However, the ependyma of young animals can be activated after injury, promoting disease progression. Moreover, neuraminidase -induced denudation of the ependymal layer also results in reactive gliosis [8]. Recently, it was found that stab injury in the brain can enhance parvalbumin (PV) expression in ependymal cells and promote their motility and adhesion, helping the scratch or the wound re-epithelialize, thereby re-establishing a continuous ependymal layer to stop the leakage of cerebrospinal fluid (CSF) [9].

In this review, we introduce the basics of ependymal cells while discussing their classification and physiological functions in the CNS in depth. At the same time, we also expounded on the important roles and future of ependymal cells from the aspects of the occurrence, development, and recovery of CNS diseases.

## 2. Subtypes and differences in ependymal cells

The heterogeneity of ependymal cells has been extensively explored. There are several different types of ependymal cells according to their different localizations, morphologies, surface markers, and functions. Gabrion and colleagues classified ependymal cells in the hypothalami or choroid plexuses in mice or rat foetuses into four groups: tanycytes, ciliated and unciliated ependymal cells, and choroidal ependymocytes [10]. Two types of tanycytes are observed in the third ventricle walls with long cytoplasmic shafts, apical blebs in the median eminence, and tight junctions. Ciliated and nonciliated ependymal cells are located in the ventrolateral walls of the third ventricle, with a lack of tightness in junctional complexes [11]. Choroidal ependymocytes are cuboidal shape with abundant long, club-shaped microvilli located in the choroid plexuses with tight junctions [10].

Another classification according to different morphologies divides ependymal cells into three types: cuboidal and radial ependymal cells and tanycytes [12]. The cuboidal ependymal cells are often arranged in a single layer that belongs to multiciliated ependymal cells with an oval nucleus and bright cytoplasm [12]. Tanycytes are often arranged in two layers, mainly existing in the median bulge and arcuate nucleus of the hypothalamus. They have irregular nuclei, dark cytoplasm, and only one type of cilium. Tanycytes are further divided into four subpopulations, α1, α2, β1, and β2, characterized by their distinct morphologies and molecular and functional features [13]. Radial ependymal cells are also arranged in two layers, with oval or irregular nuclei, bright cytoplasm, one to three types of cilia, and a long basal process [12].

**Figure 1.**
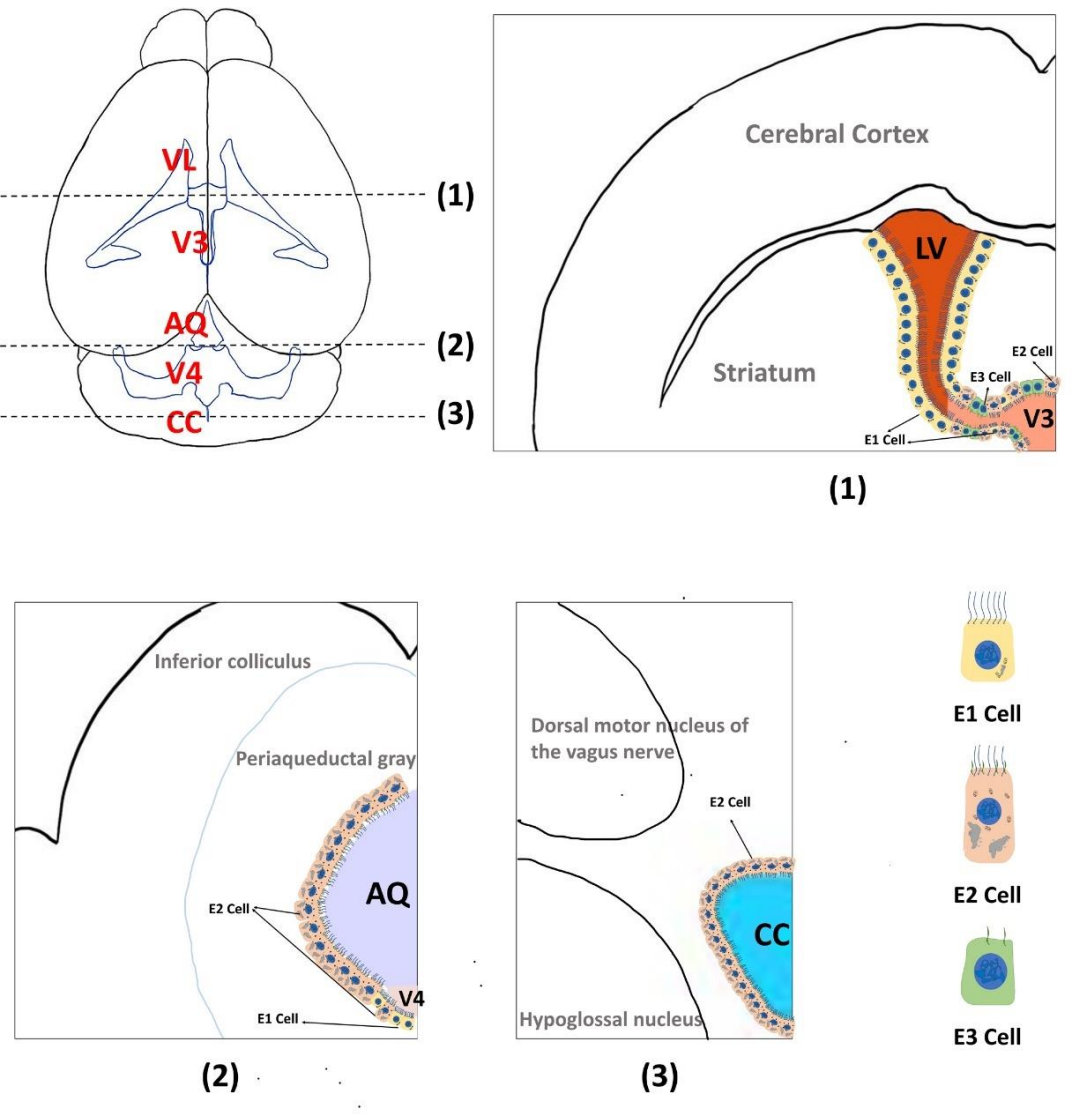
Schematic diagram of the morphology and distribution of ependymal cells. Structure of mouse ventricle (top left) from the Allen Brain Atlas with different ependymal cell subtypes in distinct ventricular regions (1) (2) (3). Ependymal cells in lateral ventricles (LV) are mainly E1 types, while E1, E2, E3 cell in V3 (1). Ependymal cells in V4 are mainly E2 types, while E1 and E2 cells in aqueduct (AQ) (2). Ependymal cells in the central canal (CC) are mainly E2 types (3). E1 type ependymal cells mainly exist in the lateral ventricle, the third and fourth ventricle of the brain, the cilia type is mainly motor cilia, and the mitochondria of E1 cells are concentrated near the basal body. E2 type ependymal cells are distributed in the spinal canal and part of the third and fourth ventricles and the cerebral aqueduct. They have both primary cilia and motor cilia, with two basal bodies surrounded by a complex set of electron density particles. E2 type ependymal cells have spherical nuclei, scattered chromatin and abundant mitochondria that concentrated around the nucleus. E3 cells are monociliated ependymal cells with only primary cilia, mainly located in the preoptic recess and infundibular recess of third ventricle.

Other studies classify ependymal cells according to different cilia numbers and locations. Cilia are slender cell protrusions that are important for the development of various organs and the maintenance of homeostasis. In human and mouse brain ventricles, ependymal cells can be categorized into three subtypes by cilia number and regional distribution (shown in Fig. 1), including multiciliated ependymal (E1 cells), ciliated ependymal (E2 cells), and uniciliated ependymal (E3 cells) [14]. Among them, the E1 type is the major ependymal cell subtype that is aligned and densely arranged from the caudal to the rostral direction and mainly exists in the lateral, third and fourth cerebral ventricles [15], playing a key role in the flow of CSF and brain homeostasis [3]. E1 cells have planar polarity, which helps the directional beating of motile cilia to maintain normal CSF flow [16]. E2 and E3 cells are responsible for collecting and transforming various extracellular signals, and β2-tanycytes in median eminence can generate newborn neurons [17]. Based on their ultrastructure, basal process, and surface marker, E2 cells in the 3 V are identified as α-tanycytes, while E3 cells in the floor of the 3 V correspond to β-tanycytes [14]. E2 cells are mainly localized in the spinal canal and part of the third and fourth ventricles and cerebral aqueduct, with both primary cilia and motile cilia [14]. The most different characteristic of E2 cells is their two large basal bodies, surrounded by a complicated set of electron-dense particles. E2 cells have a light cytoplasm, spherical nuclei, and scattered chromatin, and abundant mitochondria are concentrated around the nucleus. Mitochondria in E1 cells are confined near the basal bodies [14]. E3 cells are monociliated ependymal cells with only primary cilia, which are the 9+0 type cilia type, while both E1 and E2 ependymal cells are type 9+2 cilia. They are mainly located in the preoptic and infundibular recesses of the third ventricle [14]. Interestingly, recent single-cell RNA sequencing revealed striking homogeneity of ependymal cells across different ages, species, and regions [3], and the three major categories are still shared in all ependymal cells, including cilia-related subtype, metal ion-related subtype, and transport-related subtype. However, there remains subtle differences between the different subtypes. Neonatal ependymal cells captured a gene signature associated with developmental processes; however, adult ependymal cells, rather than ependymal cells in the canal of the spine, express cellular transport and inflammation-related genes. The molecular markers that distinguish different subtypes of ependymal cells are shown in Table 1. Moreover, E1, E2 and E3 ependymal cells are involved in cilia-related functions, ventricle shaping and metal ion regulation;E1 ependymal cells are mainly responsible for transport, but other types of ependymal cells are also reported to transport water and glycerin as they express aquaporins. It has been suggested that tanycytes are more likely to differentiate into stem cells (Table 2).

**Table 1:** Criteria for the characterization of different subtypes of ependymal cells.

	GFAP	CD24	Vimentin	S100β	Nestin	Fezf2
E1 cells	- (<16%+)	+	+	+	+	-
E2 cells	+	+	+	+	+	+
E3 cells	-	-	+	-	+	+

“+” means expression, “-”means no expression

## 3. Role of ependymal cells in the healthy CNS

The ependymal cells consist of a critical barrier. However, the function of ependymal cells remains unclear. Recently, a large number of investigations have been proformed to understand their roles in the development and physiology of the CNS. The interactions between ependymal cells and CSF and the possible role in neurogenesis have been highlighted. Ependymal cells also provide possible metabolic support for other cells. The function of ependymal cells in the healthy CNS is summarized in Table 3.

**Table 2:** Function of different types of ependymal cells.

Ependymal cell types	Functions
E1 cells	1. maintain normal CSF flow [16] 2. transport functions [3]
E2 cells	may serve as mechanical or chemical sensors of CSF flow or composition [134]
E3 cells (β-tanycytes)	1. retain a proliferative potential [14] 2. regulating progenitor activity [135] 3. sensing CSF metabolites [135]
Tanycytes	1. generate newborn neurons [135] 2. generate astrocytes [72] 3. transport bioactive substances between the CSF and blood vessSels [136]

### 3.1 Cilia-related functions

The cilium is a very important organelle with membrane-bound and centriole-derived projections from ependymal cells. It is composed of a microtubule cytoskeleton, the ciliary axoneme, encompassed by a ciliary membrane [18]. According to their ultrastructure and molecular composition, they are divided into primary cilia and motile cilia.

Primary cilia are nonmotile organelles surrounded by a membrane that is connected to the plasma membrane. Because the membrane of primary cilia has perculiar lipid and receptor content, the main function of primary cilia is to sense and transmit changes in extracellular molecules that regulate a number of intracellular signals, as well as release vesicles to modulate the function of the epithelium [19, 20]. Motile cilia, on the other hand, function as nanomachines to help cell movement [21]. Invertebrates and motile cilia participate in many physiological processes. They are mainly located in the nasal cavity [22], central canal of the spinal cord [23], and cerebral ventricles [24]. Motile cilia are also detected in the cerebral ventricular system, suggesting its role in controling CSF flow. The number of motile cilia on ependymal cells of the central canal is closely associated with the size of the spinal cord and central canal [25].

A layer of ciliated ependymocytes lines in the cerebral ventricles, contact the CSF, and they beat periodically, generating localized CSF flow [26]. Activation of the A2B receptor increases the beat frequency of ependymal cells in the lateral ventricle, which may play an essential role in controlling cerebral fluid homeostasiss [27]. It has been reported that methylmercury (MeHg), a substance that is neurotoxic to humans, inhibits the movement of ependymal cell cilia in a concentration-dependent manner and inhibited CSF flow[28]. A study in zebrafish demonstrated that CSF flow was restricted to a single ventricular cavity and directed by ependymal cilia [29]. Recently, a study showed that a CSF flow network driven by independent ependymal cilia has been formed in the early embryonic ventricular system of Xenopus, enabling CSF within and across the ventricular space [30]. The beating of ependymal cilia maintains the normal flow of CSF, which is essential for brain homeostasis, toxin clearance, convey of signalling molecules, and orientation of newborn neurons [31]. Further study showed that serotonin (5HT) and ATP were actively involved in the control of ciliary activity in ependymal cells [32].

### 3.2 Ventricle shaping

The ependymal lies at the interface between the ventricular cavities and the brain parenchyma [33]. During the first postnatal days, radial glia cells transform into type B cells and ependymal cells to constitute the subventricular zone (SVZ), but the expression or deletion of some ependymal cell-related factors effects the formation of the SVZ. For example, p73 is a key factor that controls the development of the SVZ. Depletion of P73 in ependymal cells impaired ciliogenesis and disorganized pinwheels, resulting in SVZ disruption [34]. Another factor, Camsap3, is critical for forming the lateral ventricles. Mutation of Camsap3 in mice disrupted the expansion of the apical domain of ependymal cells, resulting in narrow lateral ventricles. In addition, Camsap3 mutation obstructed extension of the neocortical VSs along the mediolateral axis [35]. Inhibitor of DNA-binding 4 protein (ID4) is involved in CNS development by controlling the proliferation and differentiation of neural stem cells. ID4 also has an effect on ventricle formation. Knockout of ID4 in mice resulted in ventricle enlargement, thinning of the ventricular wall and elongation of ependymal cells [36]. In addition, parvalbumin+ ependymal cells were found localized in the adhesions of the medial and lateral walls of the rostral LVs and showed a reactive phenotype during aging. In addition, knockout of parvalbumin ameliorated LV stenosis and adhesions in aged mice, suggesting that parvalbumin+ ependymal cells are involved in ventricle stenosis during aging [37]. Selective disruption of N-cadherin, a transmembrane glycoprotein highly expressed in the CNS, triggered massive apoptosis of ependymal cells and denudation of cerebral ventricular walls[38]. Finally, SrGAP3 deficiency enlarged lateral ventricles by 15-fold due to the reduction in the densities of cilia in ependymal cells [39].

**Table 3:** Function of ependymal cells in healthy CNS.

Function of ependymal cells in healthy CNS	Evidence
*Cilia-Related function*	1. motile cilia’ number correlates with the size of the spinal cord and central canal [25] 2. beating to maintain normal CSF flow [26]
*Ventricle shaping*	1. produced a major laminin component of the fractone bulbs in the SVZ [137]
*Transport functions*	1. E1 cells expressed transport function related genes [3] 2. involved in glucose or fructose transportation [41]
*Stem-cell like function*	1. sharing a common embryonic origin with adult neural stem cells and forming the adult neurogenic niche[55] 2. potential to turn into neuroblasts under certain environmental conditions [56] 3. generate self-renewing neurospheres and new astrocytes and oligodendrocytes [55] 4. CD9-positive ependymal cells can generate neurospheres [55] 5. regulate neural stem cells [66]

### 3.3 Transport functions

Transcriptomics data from human and mouse ventricles suggest that ependymal cells express genes related to transport functions, and E1 cells seem to be more critical in these functions [3]. The glucose transporter (GLUT) transports glucose and/or fructose in different cell types and tissues. It has been identified that GLUT1, GLUT2, GLUT5, GLUT8, and GLUT9 are expressed in ependymal cells in human and mouse brains, suggesting that ependymal cells may also participate in glucose and fructose transportation [40, 41]. GLUT1 expression is an asynchronous process with ependymal cell differentiation. Inhibition of GLUT1 results in reduced energy and angiogenesis [42]. GLUT2, as a glucose sensor in the brain, may help the hypothalamus detect changes in glucose concentration and contribute to the regulation of glucose intake as well as glucose homeostasis [43]. GLUT5, a selective transporter for D-fructose, is mainly expressed on the dorsal half of the third ventricle wall[44]. GLUT9, known as the reabsorptive urate transporter, is observed in the cilia of ependymal cells in the human brain [45]. Urate transporter 1 (URAT1), also a reabsorption urate transporter, was recently found to be expressed in cilia and the apical surface of ependymal cells [46]. Their expression in ependymal cells suggests that CSF may be involved in the urate transport system, exerting the neuroprotective function of urate.

Aquaporins (AQPs) are critical for water and glycerol transportation. AQP1, 4, 7 and 9 are expressed in ependymal cells [47]. AQP1 is largely expressed in ependymal cells at the apical membrane of the epithelium in the choroid plexus located in the lateral, third and fourth cerebral ventricles, and its main function is to produce CSF [47]. AQP has been observed surrounding brain-CSF barriers, participating in reabsorption and promoting the rapid transport of water through cell membranes [47]. Interestingly, the expression of AQP7 and AQP9 in tanycytes is related to the mouse oestrus cycle, suggesting that glycerol transport mediated by AQP7 and AQP9 can be affected by hormonal changes [48].

### 3.4 Metal ion regulations

Metal ion regulation is essential for maintaining brain homeostasis, and few studies have analysed how ependymal cells are involved in this process [3]. In Ts1Cje mice, a widely used genetic model of Down syndrome, copper transporter CTR1 expression is reduced, causing accumulation of copper in CSF and elevation of oxidative stress in the brain parenchyma [49]. Another study found that hepcidin and ferroportin are colocalized in the ependymal cells of the SVZ, indicating their regulatory function in iron export function[50]. In a rat model with elevated CNS iron loads, increased iron accumulation was found in ependymal cells, together with decreased iron levels in CSF, implying that excessive iron is actively absorbed by ependymal cells to reduce toxicity to the CNS [51].

### 3.5 Stem cell-like function

The stemness of ependymal cells has been under debate, as they seem unable to undergo sufficient self-renewal to maintain their population when injured and do not normally produce a sufficient number of other cell types [12]. It has been reported that ependymal cells and adult neural stem cells (NSCs) are sister cells, sharing a common embryonic origin and together forming the adult neurogenic niche [52]. They also share several stem cell-associated genes, overlapping transcriptional signatures, similar intracellular signalling, and some NSC protein ‘markers’, such as Sox2, Nestin, SRY-Box2, and CD133 [52-55]. In the adult lateral ventricle wall, ependymal cells show high expression of many genes controlled by the transforming growth factor-β family, which indicates their potential to transform into neuroblasts [56]. The stemness of ependymal cells seems to appear in certain locations of the brain, especially in the subependymal, central canal of the spinal cord, ventriculus terminalis, and choroid plexus ependymal regions [57-60]. CD133/CD241/CD45/CD34 ependymal cells isolated from the spinal cord can form neurospheres under cloning conditions and differentiate into three neural lineages [56]. In the central canal, ependymal cells can generate proliferative neurospheres and differentiate into astrocytes and oligodendrocytes [61]. In addition, they can acquire stem cell properties after injury or stimulation. Ependymal cell dedifferentiation can be induced by IKK2 inhibitors [62]. After spinal cord injury (SCI), 95% of reactive ependymal cells differentiate into astrocytes (mostly) or oligodendrocytes and form glial scars [12]. High mobility group Box 1 (HMGB1) secreted from reactive astrocytes promoted this process, and inhibited ependymal cell differentiation into neurons [63]. CD9-positive ependymal cells in adult rats can generate neurospheres [55]. Ependymal cells in the adult spinal cord (SCEp cells) rarely proliferate under normal conditions; however, overexpression of the Notch intracellular domain in SCEp cells increases their proliferation and promotes their differentiation into astrocytes [52]. An in vivo study demonstrated that a certain number of SCEp cells differentiated into astrocytes after grafting into the spinal cord [60]. After brain damage in the medulla oblongata, tanycytes are a subtype of ependymal cells that can provide new neuronal lineage cells [55]. Ependymal cells can also function as neural stem-like cells to rebuild the neural network during chordate metamorphosis [64].

In some cases, ependymal cells contribute to neurogenesis by releasing factors or signals rather than differentiating into stem cells. The ependymal cells that capulate the stem cell niche express LRP2, the receptor of BMP4 to mediate BMP4 catabolism, while LRP2 deficiency inhibits neural precursor cell proliferation and neuroblast migration to the olfactory bulb [65]. The choroid plexus-derived ependymal cells grafted into spinal cord lesions secrete trophic factors and interact with growing axons via adhesion molecules, supporting nerve regeneration in the spinal cord [66].

Ependymal cells and neural progenitor cells share a common origin and many molecular markers [52, 55]. As early as 1996, it was reported that the presence of cells in the ventricle can generate neurospheres and differentiate into glial cells, and it was speculated that it might be derived from ependymal cells in the ventricle [67]. Lineage tracing studies suggest that after spinal cord injury, most stem cells are derived from ependymal cells, which can differentiate into glial cells [12, 68]. For example, ependymal cells migrate from the ependymal layer to the lesion and differentiate into astrocytes after spinal cord injury [69]. To date, many studies have proposed the stemness of ependymal cells in the CNS after injury; however, the mechanism still needs to be explored.

### 3.6 Sleep and neurogenesis regulation

The thalamus is considered to be a key structure involved in mediating circadian rhythms [20]. According to the location and function of ependymal cells, researchers suggest that ependymal cells may play a role in regulating sleep. For example, IL-18 secreted by ependymal cells was able to modulate sleep and influence long-term potentiation [70]. Fatty acid amide hydrolase derived from ventricular ependymal cells can also modulate sleep [71].

A local neural stem/progenitor cell niche exists in the adult hypothalamus, in which α2-tanycytes is a key component, and α1 and β-tanycytes have limited self-renewal capacity. α2-tanycytes regulate their proliferation through fibroblast growth factor signaling, maintain self-renewal, or generate β-tanycytes and parenchymal astrocytes, and even a small number of neurons [72].

## 4. Role of ependymal cells in CNS diseases

Under certain pathological conditions, ependymal cells change their morphology and function through a variety of signalling pathways. Here we describe the important roles and future development of ependymal cells in the occurrence, development, and recovery of CNS diseases such as cerebrovascular disease, spinal cord injury, hydrocephalus, and mental disease. The function of ependymal cells in the CNS diseases is summarized in Table 4.

**Table 4:** Function of ependymal cells in CNS diseases.

Disease	Morphological changes of ependymal cells	Molecules involved in the disease
*Stroke*	1. Almost lost in major stroke [76, 80]. 2. Became less cuboid, flattened and stretched in scar formation and surface area was increased [78]. 3. Motor cilia and basal bodies were displaced in the anterior dorsal SVZ [76]. 4. Non-stem- or progenitor-like responses [80].	1. Expressed robust levels of GFAP in 7 and 14 days after stroke [76, 81]. 2. Notch signaling as the regulator [77]. 3. Up-regulated the expression of the O-linked N-acetylglucosamine containing epitope H [82].
*Spinal cord injury (SCI)*	1. Act as a potential repair cell source [87, 92]. 2. Became multilineage differentiation [12, 91].	1. Nestin-positive cells are increased [88, 125]. 2.BAF45D expression is reduced [87]. 3. Intracellular Ca2+ level is increased [89]. 4. Interleukin-17A is a negative regulator [94].
*Hydrocephalus*	1. Cilia lack the coordinated orientation [100]. 2. Ependymal cell desquamation and subependymal basal membrane destruction [79].	1. Daple-deficient, Mpdz-deficient, RFX3-deficient, MT1-MMP-deficient, SNX27-deficient [98, 99, 109, 111]. 2.VPS35 prevents neonatal hydrocephalus [103]. 3. Inactivation of Cdc42 lead to hydrocephalus [108].
*Psychiatric*	1. Ependymal planar cell polarity is disrupted [138]. 2. CSF flow is reduced [138].	1. P11-deficient [86]. 2. Ubiquitin-positive inclusions were found [126].
*Brain injury*	1.PV+ ependymal cells promote wound-healing [9]. a positive correlation between the percentage of nestin+ ependymal cells and post-injury survival time in mice after brain trauma [125].	1. Up-regulation of calcium-binding protein parvalbumin-expression [9].
*Acute aseptic neuroinflammation*	1. Impaired ependymal cell viability and even cell death [127].	Not mentioned.
*Aging*	1. The ependymal layer thins [54]. 2. The density of apical motional cilia is reduced [54]. 3. Accumulate the lipid droplets [54].	Not mentioned.
*Alzheimer's disease*	1. Lipid droplets rich in oleic acid side chains selectively accumulate in the ependymal cells [54].	Not mentioned.
*Age-related ventricular stenosis*	1. p73 knock-in mouse showed aqueductal stenosis, p73 knock out mouse showed aqueductal stenosis at a later stage but not hydrocephalus	1. p73 [139].

### 4.1 Roles of ependymal cells in cerebrovascular disease

Stroke, one of the most common cerebrovascular diseases, is the leading cause of morbidity and mortality worldwide [73]. Usually, in the lesion area of stroke, microglia and astrocytes are extensively studied [74, 75] while ependymal cells are almost completely lost and most of them do not proliferate or divide too slowly to be observed [76, 77]. After stroke, the number of ependymal cells decreased in the neurogenic niche at 6 and 16 weeks, and ectopic ependymal cells migrated to the striatum of the stroke brain and were involved in scar formation[78]. Their ultrastructural properties changed and became less cuboid, flattened, and stretched[78]. The cell surface area was also increased; moreover, destruction of the ependymal cilia was also observed. The ependymal motor cilia and basal bodies were displaced in the anterior dorsal SVZ, disrupting the ependymal planar cell polarity, ultimately resulting in slower and more turbulent CSF flow [76]. Subarachnoid haemorrhage causes desquamation of choroidal artery vasospasm-related ependymal cells and rupture of the subependymal basal membrane [79]. After stroke, ependymal cells showed nonstemness [80]. Ependymal cells expressed robust levels of GFAP in the brain ipsilateral to the lesion and exhibited the phenotype and morphology of radial glia and reactive astrocytes at 7 and 14 days after stroke[76, 81]. Ependymal cells in the forebrain generate neuroblasts and astrocytes, which are regulated by Notch signalling. However, ependymal cells have difficulty maintaining their population by self-renewal and are reduced when generating differentiating progeny; thus, they are more likely to serve as a source that is stimulated by injury rather than serving as stem cells [77]. In human ependymal cells, hypoxia upregulated the level of the O-linked N-acetylglucosamine-containing epitope H [82].

### 4.2 Ependymal cells in psychiatric disorder

As the number of major depressive disorder (MDD) patients continues to increase, increasing attention has been given to this disease. However, the underlying mechanism of MDD remains unclear. Depression is not only a neurological disease with anxiety disorder but also closely associated with a variety of chronic and senile diseases, making the prognosis complicated and aggravating the situation of disease and disability in the world [83]. Ependymal cells play critical roles in propelling the CSF, and interference with ependymal planar cell polarity leads to aberrant CSF circulation, which may influence neurodegenerative and psychiatric diseases [1]. P11 (S100A10) is an important molecule that regulates the aetiology of depression [84, 85]. A previous study demonstrated that patients with MDD and a chronic stress-induced mouse depression model showed decreased levels of p11, caused by disruption of ependymal planar cell polarity and reduction of CSF flow, resulting in depression and anxiety [86].

### 4.3 Ependymal cells in spinal cord injury

Ependymal cells have been studied extensively in SCI. After SCI, ependymal cells in the central canal of the spinal cord (SCECs) proliferate, differentiate, and migrate to the lesion site and act as a potential repair cell source [87]. The proliferation and differentiation of ependymal cells vary according to the severity of the injury. SCI induced the proliferation of nestin+ ependymal cells, which is positively correlated with the survival time of mice [88]. In SCECs after SCI, the level of BAF45D is reduced, which may inhibit nerve growth [87]. In an ex vivo SCI model, ependymal cells are activated, proliferate, migrate out of the central canal, differentiate and show spontaneous Ca^2+^ activity and are responsive to ATP with increasing intracellular Ca2+ levels[89]. Cultured choroid plexus ependymal cells transplanted to the spinal cord can transform into astrocytes[90]. After SCI, ependymal cells showed multilineage differentiation, mainly differentiating into astrocyte-like cells and to a lesser degree, oligodendrocytes. Ependymal cells migrate to the injury site to constitute the glial scar and even support local sprouting [12, 91]. In vivo juvenile ependymal cells contribute more to glial scar formation and show a higher recruitment potential[92]; however, some investigators claimed that their contribution to scar formation is local, primarily in the vicinity of the damaged area, and dependent directly on ependymal injury [93]. In these processes, interleukin-17A(IL-17A) is negatively associated with ependymal cell proliferation and suppressing IL-17A in ependymal cells promotes neurotrophic factor levels and axonal sprouting in the injured spinal cord [94].

### 4.4 Ependymal cells in hydrocephalus

Broadly speaking, hydrocephalus is defined as any increase in CSF, including brain oedema. In a narrow sense, it is defined as ventricular enlargement that accelerates head growth or requires surgical intervention [95]. Acute hydrocephalus is a common complication after spontaneous subarachnoid haemorrhage (SAH) that severely threatens life. After SAH, the frequency of occurrence of hydrocephalus ranges from 15% to 20%. Impairment of CSF and reduced absorption secondary to fibrosis of the leptomeninges and arachnoid granulations are considered causes of hydrocephalus after SAH[96]. Therefore, ependymal cells may have a certain connection with hydrocephalus. After SAH, aqueductal stenosis and hydrocephalus may be caused by desquamation of ependymal cells and disruption of the subependymal basal membrane [79]. A series of molecules in ependymal cells may be link to hydrocephalus. Knockout of Daple, a Dvl-associated protein with high leucine content (Ccdc88c), caused hydrocephalus and disorientation of ependymal cilia [97, 98]. The assembly and function of cilia in nematodes, Drosophila and mice are regulated by the regulatory Factor X (RFX) family [99]. It has been demonstrated that knockout of RFX3 in mice caused hydrocephalus but not stenosis of the Sylvius aqueduct, which is characterized by hypoplasia of secretory ependymal cells of the subcommissural organ and changes in the number rather than ultrastructure of cilia [100, 101]. Vacuolar protein sorting-associated protein 35 (VPS35) is involved in the retrieval of transmembrane proteins or cargos from endosomes to the trans-Golgi network or recycling cargos from endosomes to the cell surface [102]. A recent study suggested that Vps35 not only promotes the differentiation of ependymal cells in a cell-autonomous manner but also inhibits microglial activation, radial glial cell or ependymal precursor cell proliferation and death in a cell nonautonomous manner, thus preventing neonatal hydrocephalus [103]. Adherens junctions (AJs) play a critical role in connecting ependymal cells to maintain tissue structure integrity. Afadin, an actin filament-binding protein, is crucial for maintaining AJs in ependymal cells by binding to nectins and acatenin [104, 105], which is required for the development of the cerebral aqueduct and ventral third ventricle in the midbrain [106]. Another important molecule is Cdc42, a small GTPase that regulates many cellular functions, especially cell polarity [107]. Inactivation of Cdc42 leads to hydrocephalus, causes death during the postnatal stage and disrupts ependymal cell differentiation, resulting in aqueductal stenosis [108]. Membrane-type 1-MMP (MT1-MMP) has been shown to be a regulator of ependymal cells. Jiang et al. reported that knockout of MT1-MMP decreased and disrupted motile cilia and impaired ependymal cell maturation, causing abnormal CSF flow and hydrocephalus [109]. To date, two genes responsible for causing nonsyndromic congenital hydrocephalus in humans have been detected, and MPDZ is one of them [110]. MPDZ deficiency reduced the interacting planar cell polarity protein Pals1 in ependymal cells, accompanied by the loss of barrier integrity and reactive astrogliosis, ultimately resulting aqueductal stenosis [111]. SNX27 is an endosomal sorting factor highly expressed in the brain and has been suggested to participate in the trafficking of multiple transmembrane receptors. Knockout of SNX27 reduced the number of ependymal cells and cilia density, leading to severe postnatal hydrocephalus [112].

### 4.5 Ependymal cells in multiple sclerosis

Multiple sclerosis is a chronic demyelinating disease that damages the spinal cord and brain, resulting in neuronal loss and brain atrophy. The periventricular is one of the main pathological changes in multiple sclerosis, and researchers found that the flow of cerebrospinal fluid is abnormal after MS, and MR results showed that ependymal cells are damaged after MS [113, 114]. Experimental animals with ependymal-related gene knockout have pathological changes similar to multiple sclerosis, such as periventricular demyelination, brain atrophy, and ventricular enlargement, indicating that there is a link between ependymal cells and multiple sclerosis. However, the underlying mechanism remains unknown [115]. In addition, the entry of myelin debris into the cerebrospinal fluid can also cause damage and apoptosis of ependymal cells [116]. In addition, several articles have reported that ependymal cells express MS-related cytokines [53, 117, 118]. The correlation between MS and ependymal cells still needs to be investigated.

### 4.6 Ependymal cells in aging and Alzheimer's disease

Aging results in the loss of ependymal cells and increases the proliferation of reactive astrocytes. In addition, aging causes thinning of the ependymal layer, reduction of the density of apical motional cilia, and accumulation of lipid droplets in ependymal cells. In agingIn age-related ventricular stenosis, ependymal cells are damaged, and PV is upregulated [37]. Metabolic and barrier functions of ependymal cells significantly decline during aging in mice. These functions partially depend on the expression of myristoylated alanine-rich protein kinase C substrate (MARCKS), and reduction of MARCKS during aging leads to intracellular mucin accumulation, oxidative stress, and lipid droplet accumulation, resulting in disruption of the ependymal barrier function [119].

In Alzheimer's disease, irregular and thickening basement membranes can also be observed, similar to to aging [120, 121]. Biondi ring tangles, lipofuscin deposits and lipid droplets rich in oleic acid side chains selectively accumulate in the ependymal cells, ultimately leading to the deterioration of the neurogenic niche of the SVZ, further suppressing neurogenesis and destroying striatal and hippocampal function [54]. Aquaporins such as AQP1 expression and CSF secretion were also diminished [122, 123]. By using an unbiased proteomic analyses on autopsy tissue from AD, 25 proteins were found to be significantly increased on the lateral ventricle walls, including amyloid-β, indicating abnormalities in the clearance of some toxic molecules and a decrease in CSF turnover around the ependyma in AD pathology [124].

### 4.7 Ependymal cells in other neurological disorders

There are connections between ependymal cells and other brain diseases. Inflammatory cytokines released from brain injury upregulate parvalbumin (PV) expression, which promotes the motion and adhesion of ependymal cells and improves ependymal layer re-establishment and leakage closure[9]. In the cervical spinal cord sections of 27 patients who died from severe trauma to the CNS, there was a significant increase in nestin-positive ependymal cells, which was positively correlated with postinjury survival time [125], indicating that some ependymal cells have the potential for neurogenesis in response to brain injury. Ubiquitin-positive inclusions (UbIs) were found in ependymal cells, especially in the central canal, and central ependymal UbIs are more frequent in patients with neurological disorders, although they are not disease-specific [126]. Additionally, in a rat model of acute aseptic neuroinflammation, microglia activate, and ependymal cell viability was impaired and even led to ependymal cell death [127].

### 4.8 Interaction between ependymal cells and other cells

**Figure 2.**
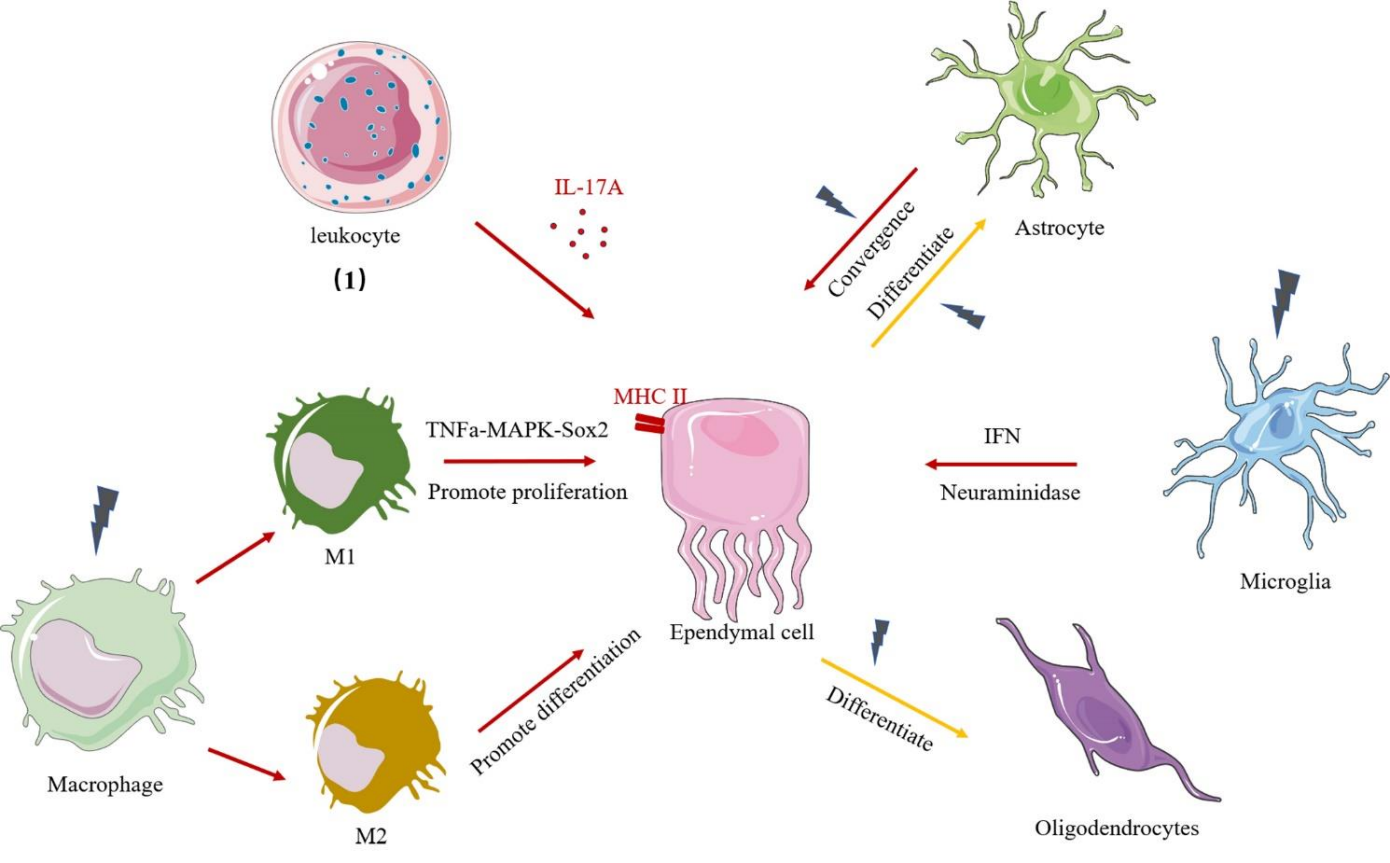
Ependymal cells interact with glia cells and immune cells. (1)-(2) Ependymal cells express MHC II antigen and interact with immune cells. (1) Leukocytes affect ependymal cells via IL-17A. (2) Macrophages are activated to M1 or M2 macrophages after central nervous system injury. M1 macrophages promote the proliferation of ependymal cells, and M2 macrophages promote ependymal cell differentiation. (3)-(5) Ependymal cells interact with glia cells. (3) Astrocytes exhibit ependymal-like phenotype and function after central nervous system injury. Ependymal cells exhibit stem-like differentiation into astrocytes after CNS injury. (4) Microglia damage ependymal cells via neuraminidase and IFN pathways after CNS injury. (5) Ependymal cells exhibit stem-like differentiation into oligodendrocytes after CNS injury.

Previous studies have shown that ependymal cells interact with astrocytes, microglia, and immune cells (Fig. 2). It has been demonstrated that injection of the enzyme neuraminidase derived from Clostridium perfringens into the lateral ventricle activated microglia, astrocytes, and immune cells, including neutrophils, macrophages, T cells and B cells, infiltrating the brain. Activated microglia are involved in ependymal damage and cause hydrocephalus through IL-1β [128, 129]. Other studies reported that conditional knockout of Vps35 in ependymal cells activated microglia and caused hydrocephalus, while depletion of microglia rescued the pathology of hydrocephalus [103].

In addition to microglia, ependymal cells closely interact with astrocytes to modulate CNS function. Ependymal cells and astrocytes belong to the radial glia during development [2]. Thus, many studies have shown that ependymal cells can differentiate into astrocytes after CNS injury [12, 68, 77, 130, 131]. Lattke et al. reported that NF-kB activation in astrocytes impaired ependymal cilia formation and led to hydrocephalus [132].

After spinal cord injury, CD45+ immune cells modulate ependymal cell proliferation via IL17A, and inhibition of IL17A increases ependymal cell proliferation and functional recovery [94]. Ma and colleagues showed that M1 macrophages increased the proliferation of ependymal cells via TNFa-MAPK-Sox2 signalling, while M2 macrophages promoted neuronal differentiation of ependymal cells [133].

## 5. Summary and conclusions

As an essential part of the nervous system, ependymal cells separate nerve tissue from CSF and serve as a bridge between the brain/spinal cord and CSF. Their unique location makes them an important structure involved in the regulation mechanisms of both nerve and cerebrospinal fluid, and their function has been verified in a variety of neurological diseases. The healthy circulation of CSF is critical for maintaining homeostasis in the brain, and ependymal cells can either secrete substances to CSF or sense changes in CSF by direct contact, transmitting information to neural tissues. In addition, while controversial, ependymal cells have been identified in certain sites with stem cell characteristics. This effect is exhibited after SCI, highlighting them as potential nerve regeneration targets. It is of great importance to study the potential and properties of ependymal cells to modulate their response to CNS injury damage. Overall, the function of ependymal cells remains unclear, but due to their specific physical location in the brain and their diverse cellular functions, they can serve as a new target for neural regeneration.
